# (Benzene-1,3-dicarboxyl­ato-κ^2^ *O* ^1^,*O* ^1′^)(1,12,15,26-tetra­aza-5,8,19,22-tetra­oxa-3,4:9,10:17,18:23,24-tetra­benzocyclo­octa­cosane-κ^4^ *N* ^1^,*N* ^12^,*N* ^15^,*N* ^26^)cadmium(II) benzene-1,3-dicarboxylic acid solvate

**DOI:** 10.1107/S1600536808033138

**Published:** 2008-10-18

**Authors:** Zhi-Fang Jia, Jian-Fang Ma, Lai-Ping Zhang

**Affiliations:** a Department of Chemistry, Northeast Normal University, Changchun 130024, People’s Republic of China

## Abstract

In the title compound, [Cd(C_8_H_4_O_4_)(C_36_H_44_N_4_O_4_)]·C_8_H_6_O_4_, the Cd^II^ atom is six-coordinated by four N atoms from the macrocyclic ligand and two O atoms from a benzene-1,3-dicarboxyl­ate ligand. The complex mol­ecules are linked by N—H⋯O hydrogen bonds, forming a one-dimensional chain structure along the *b* axis. The chains are further connected through N—H⋯O and O—H⋯O hydrogen bonds between the complex mol­ecule and an uncoordinated benzene-1,3-dicarboxylic acid mol­ecule, resulting in a two-dimensional supra­molecular network.

## Related literature

For general background, see: Banerjee *et al.* (2005 ▶); Liu *et al.* (2005 ▶). For a related structure, see: Sarkar *et al.* (2008 ▶).
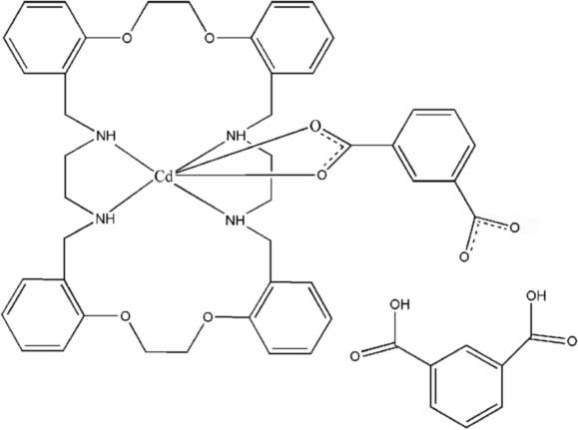


## Experimental

### 

#### Crystal data

[Cd(C_8_H_4_O_4_)(C_36_H_44_N_4_O_4_)]·C_8_H_6_O_4_
*M* *_r_* = 1039.39
Monoclinic, 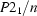

*a* = 12.912 (3) Å
*b* = 23.330 (5) Å
*c* = 16.086 (3) Å
β = 103.37 (3)°
*V* = 4714.4 (18) Å^3^
*Z* = 4
Mo *K*α radiation
μ = 0.53 mm^−1^
*T* = 293 (2) K
0.34 × 0.29 × 0.21 mm

#### Data collection

Rigaku R-AXIS RAPID diffractometer
Absorption correction: multi-scan (*ABSCOR*; Higashi, 1995 ▶) *T*_min_ = 0.842, *T*_max_ = 0.913
43452 measured reflections
10582 independent reflections
4951 reflections with *I* > 2σ(*I*)
*R*_int_ = 0.158

#### Refinement

*R*[*F* ^2^ > 2σ(*F* ^2^)] = 0.085
*wR*(*F* ^2^) = 0.185
*S* = 1.02
10582 reflections
640 parameters
9 restraints
H atoms treated by a mixture of independent and constrained refinement
Δρ_max_ = 0.68 e Å^−3^
Δρ_min_ = −0.71 e Å^−3^

### 

Data collection: *PROCESS-AUTO* (Rigaku, 1998 ▶); cell refinement: *PROCESS-AUTO*; data reduction: *PROCESS-AUTO*; program(s) used to solve structure: *SHELXS97* (Sheldrick, 2008 ▶); program(s) used to refine structure: *SHELXL97* (Sheldrick, 2008 ▶); molecular graphics: *SHELXTL* (Sheldrick, 2008 ▶) and *DIAMOND* (Brandenburg, 1999 ▶); software used to prepare material for publication: *SHELXL97*.

## Supplementary Material

Crystal structure: contains datablocks global, I. DOI: 10.1107/S1600536808033138/hy2157sup1.cif

Structure factors: contains datablocks I. DOI: 10.1107/S1600536808033138/hy2157Isup2.hkl

Additional supplementary materials:  crystallographic information; 3D view; checkCIF report

## Figures and Tables

**Table 1:** Selected geometric parameters (Å, °)

Cd1—N1	2.433 (6)
Cd1—N2	2.321 (6)
Cd1—N3	2.329 (6)
Cd1—N4	2.374 (6)
Cd1—O11	2.302 (6)
Cd1—O12	2.462 (6)

**Table 2:** Hydrogen-bond geometry (Å, °)

*D*—H⋯*A*	*D*—H	H⋯*A*	*D*⋯*A*	*D*—H⋯*A*
N3—H3N⋯O8	0.82 (5)	2.42 (4)	3.148 (8)	147 (6)
N4—H4N⋯O4	0.85 (6)	2.46 (6)	3.043 (8)	127 (6)
N2—H2N⋯O9^i^	0.83 (2)	2.10 (3)	2.901 (7)	163 (6)
O8—H8O⋯O9^i^	0.87 (2)	1.76 (3)	2.605 (7)	166 (9)
O5—H5O⋯O10^ii^	0.85 (8)	1.71 (8)	2.548 (9)	170 (12)

Symmetry codes: (i) 
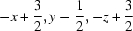
; (ii) 
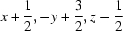
.

**Fig. 1.**
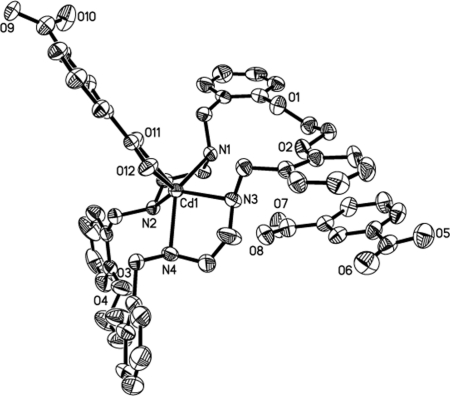
Molecular structure of the title compound. Displacement ellipsoids are drawn at the 30% probability level. H atoms have been omitted for clarity.

**Fig. 2.**
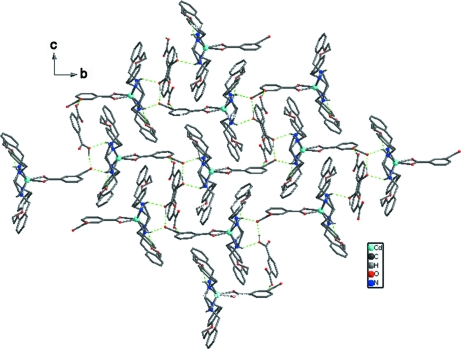
View of the two-dimensional supramolecular structure of the title compound. Hydrogen bonds are shown as dashed lines.

## supplementary crystallographic information

### Comment

As part of an investigation of the supramolecular chemistry of crown ether systems, we present here the crystal structure of the title compound.

In the title compound, the Cd^II^ atom displays a psudo-square-pyramidal coordination geometry, defined by four N atoms from the macrocyclic ligand in the basal plane and two O atoms of the carboxylate group of a benzene-1,3-dicarboxylate ligand (Fig. 1). The bond distances (Cd—N, Cd—O) and angles (N—Cd—N, N—Cd—O) are normal (Table 1) (Banerjee *et al.*, 2005; Liu *et al.*, 2005). The complex molecules are linked by N—H···O hydrogen bonds, forming a one-dimensional chain structure along the *b*-axis (Table 2). The uncoordinated benzene-1,3-dicarboxylic acid molecules are located on both sides of the chain. The adjacent chains are further connected through N—H···O and O—H···O hydrogen bonds between the complex molecule and the benzene-1,3-dicarboxylic acid, resulting in a two-dimensional supramolecular network (Fig. 2) (Sarkar *et al.*, 2008).

### Experimental

A mixture of Cd(OH)_2 _(0.146 g, 1 mmol), benzene-1,3-dicarboxylic acid (0.162 g, 1 mmol) and 3,4:9,10:17,18:23,24-tetrabenzo-1,12,15,26-tetraaza-5,8,19,22- tetraoxacyclooctacosane (0.596 g, 1 mmol) in EtOH (10 ml) was placed in a 20 ml Teflon-lined reactor and heated at 393 K for 3 d. After the reactor was gradually cooled to room temperature at a rate of 10 K h^-1^, colorless crystals were obtained.

### Refinement

H atoms bound to C atoms were positioned geometrically and refined as riding, with C—H = 0.93 (CH) and 0.97 Å (CH_2_) and with *U*_iso_(H) = 1.2*U*_eq_(C). H atoms bound to N and O atoms were located in a difference Fourier map and refined with restraints of N—H = 0.84 (2) and O—H = 0.86 (2) Å and with *U*_iso_(H) = 1.2*U*_eq_(N) or *U*_iso_(H) = 1.5*U*_eq_(O).

### Crystal data

**Table:**

[Cd(C_8_H_4_O_4_)(C_36_H_44_N_4_O_4_)]·C_8_H_6_O_4_	*F*(000) = 2152
*M**_r_* = 1039.39	*D*_x_ = 1.464 Mg m^−^^3^
Monoclinic, *P*2_1_/*n*	Mo *K*α radiation, λ = 0.71073 Å
Hall symbol: -P 2yn	Cell parameters from 10582 reflections
*a* = 12.912 (3) Å	θ = 3.1–27.5°
*b* = 23.330 (5) Å	µ = 0.53 mm^−^^1^
*c* = 16.086 (3) Å	*T* = 293 K
β = 103.37 (3)°	Block, colourless
*V* = 4714.4 (18) Å^3^	0.34 × 0.29 × 0.21 mm
*Z* = 4	

### Data collection

**Table:**

Rigaku R-AXIS RAPID diffractometer	10582 independent reflections
Radiation source: 18 kW rotation anode	4951 reflections with *I* > 2σ(*I*)
graphite	*R*_int_ = 0.158
ω scans	θ_max_ = 27.5°, θ_min_ = 3.1°
Absorption correction: multi-scan (ABSCOR; Higashi, 1995)	*h* = −16→15
*T*_min_ = 0.842, *T*_max_ = 0.913	*k* = −30→30
43452 measured reflections	*l* = −20→20

### Refinement

**Table:**

Refinement on *F*^2^	Primary atom site location: structure-invariant direct methods
Least-squares matrix: full	Secondary atom site location: difference Fourier map
*R*[*F*^2^ > 2σ(*F*^2^)] = 0.085	Hydrogen site location: inferred from neighbouring sites
*wR*(*F*^2^) = 0.185	H atoms treated by a mixture of independent and constrained refinement
*S* = 1.02	*w* = 1/[σ^2^(*F*_o_^2^) + (0.0557*P*)^2^ + 6.3548*P*] where *P* = (*F*_o_^2^ + 2*F*_c_^2^)/3
10582 reflections	(Δ/σ)_max_ = 0.001
640 parameters	Δρ_max_ = 0.68 e Å^−^^3^
9 restraints	Δρ_min_ = −0.71 e Å^−^^3^

### Special details

**Table:**

Refinement. The crystal quality is not fine and weakly diffracting, so that the value of *R*(int) is greater than 0.15.

### Fractional atomic coordinates and isotropic or equivalent isotropic displacement parameters (Å^2^)

**Table:**

	*x*	*y*	*z*	*U*_iso_*/*U*_eq_	
Cd1	0.82429 (4)	0.70844 (2)	0.68005 (3)	0.04757 (17)	
C1	0.7605 (6)	0.6571 (3)	0.9444 (4)	0.060 (2)	
C2	0.6668 (8)	0.6673 (4)	0.9702 (6)	0.090 (3)	
H2	0.6228	0.6975	0.9463	0.108*	
C3	0.6387 (9)	0.6330 (7)	1.0308 (7)	0.126 (5)	
H3	0.5769	0.6406	1.0492	0.151*	
C4	0.7012 (12)	0.5884 (6)	1.0634 (6)	0.122 (5)	
H4	0.6794	0.5639	1.1016	0.147*	
C5	0.7954 (11)	0.5784 (5)	1.0419 (6)	0.109 (4)	
H5	0.8402	0.5490	1.0675	0.131*	
C6	0.8223 (8)	0.6133 (4)	0.9807 (5)	0.074 (2)	
C7	1.0047 (8)	0.5921 (5)	1.0100 (6)	0.116 (4)	
H7A	0.9959	0.5561	1.0378	0.140*	
H7B	1.0177	0.6217	1.0535	0.140*	
C8	1.1002 (8)	0.5876 (5)	0.9714 (7)	0.116 (4)	
H8A	1.1646	0.5853	1.0165	0.140*	
H8B	1.0951	0.5532	0.9370	0.140*	
C9	1.1934 (7)	0.6434 (4)	0.8896 (6)	0.088 (3)	
C10	1.2900 (8)	0.6155 (5)	0.9199 (6)	0.115 (4)	
H10	1.2977	0.5887	0.9636	0.138*	
C11	1.3740 (9)	0.6284 (6)	0.8837 (9)	0.125 (5)	
H11	1.4389	0.6099	0.9030	0.150*	
C12	1.3626 (8)	0.6679 (5)	0.8201 (9)	0.118 (4)	
H12	1.4198	0.6770	0.7964	0.142*	
C13	1.2657 (7)	0.6944 (4)	0.7910 (6)	0.090 (3)	
H13	1.2587	0.7213	0.7474	0.108*	
C14	1.1804 (6)	0.6827 (3)	0.8234 (5)	0.068 (2)	
C15	1.0722 (5)	0.7088 (3)	0.7890 (4)	0.0609 (18)	
H15A	1.0460	0.7242	0.8363	0.073*	
H15B	1.0793	0.7405	0.7516	0.073*	
C16	1.0233 (7)	0.6366 (4)	0.6768 (5)	0.112 (4)	
H16A	0.9934	0.5984	0.6763	0.134*	
H16B	1.1002	0.6326	0.6917	0.134*	
C17	0.9913 (7)	0.6604 (5)	0.5876 (5)	0.109 (4)	
H17A	1.0314	0.6952	0.5845	0.131*	
H17B	1.0097	0.6329	0.5480	0.131*	
C18	0.8618 (6)	0.7140 (3)	0.4908 (4)	0.0618 (19)	
H18A	0.9104	0.7459	0.5070	0.074*	
H18B	0.7899	0.7289	0.4809	0.074*	
C19	0.8784 (6)	0.6886 (3)	0.4090 (4)	0.060 (2)	
C20	0.9703 (7)	0.6970 (4)	0.3810 (5)	0.078 (2)	
H20	1.0252	0.7175	0.4161	0.094*	
C21	0.9863 (8)	0.6769 (4)	0.3043 (6)	0.088 (3)	
H21	1.0501	0.6830	0.2882	0.106*	
C22	0.9040 (9)	0.6474 (4)	0.2527 (5)	0.089 (3)	
H22	0.9115	0.6337	0.2001	0.107*	
C23	0.8087 (8)	0.6377 (4)	0.2782 (5)	0.079 (3)	
H23	0.7531	0.6180	0.2426	0.095*	
C24	0.7984 (6)	0.6576 (3)	0.3560 (4)	0.061 (2)	
C25	0.6174 (6)	0.6254 (3)	0.3339 (5)	0.069 (2)	
H25A	0.6326	0.5885	0.3115	0.083*	
H25B	0.5930	0.6513	0.2862	0.083*	
C26	0.5341 (6)	0.6190 (3)	0.3835 (5)	0.071 (2)	
H26A	0.4684	0.6056	0.3463	0.085*	
H26B	0.5567	0.5910	0.4286	0.085*	
C27	0.4366 (6)	0.6752 (3)	0.4613 (5)	0.062 (2)	
C28	0.3364 (6)	0.6499 (3)	0.4314 (5)	0.071 (2)	
H28	0.3222	0.6279	0.3819	0.086*	
C29	0.2600 (6)	0.6585 (4)	0.4766 (7)	0.088 (3)	
H29	0.1943	0.6406	0.4583	0.105*	
C30	0.2770 (7)	0.6917 (4)	0.5464 (7)	0.095 (3)	
H30	0.2229	0.6979	0.5747	0.115*	
C31	0.3760 (7)	0.7167 (4)	0.5760 (5)	0.080 (3)	
H31	0.3876	0.7399	0.6242	0.096*	
C32	0.4574 (6)	0.7078 (3)	0.5354 (4)	0.0611 (19)	
C33	0.5690 (5)	0.7290 (3)	0.5729 (4)	0.061 (2)	
H33A	0.5943	0.7499	0.5293	0.073*	
H33B	0.5674	0.7554	0.6191	0.073*	
C34	0.6087 (6)	0.6435 (3)	0.6662 (4)	0.064 (2)	
H34A	0.6400	0.6059	0.6645	0.077*	
H34B	0.5319	0.6394	0.6492	0.077*	
C35	0.6395 (5)	0.6666 (3)	0.7562 (4)	0.0595 (19)	
H35A	0.6105	0.7049	0.7574	0.071*	
H35B	0.6090	0.6425	0.7934	0.071*	
C36	0.7893 (6)	0.6938 (3)	0.8745 (4)	0.060 (2)	
H36A	0.7562	0.7311	0.8744	0.072*	
H36B	0.8658	0.6995	0.8878	0.072*	
C38	0.7114 (7)	0.5151 (4)	0.5534 (5)	0.071 (2)	
C39	0.7151 (6)	0.4983 (3)	0.4656 (5)	0.0593 (19)	
C40	0.6242 (6)	0.4738 (3)	0.4124 (5)	0.072 (2)	
H40	0.5629	0.4677	0.4322	0.086*	
C41	0.6280 (8)	0.4590 (4)	0.3298 (5)	0.086 (3)	
H41	0.5688	0.4422	0.2941	0.104*	
C42	0.7189 (8)	0.4687 (3)	0.2990 (6)	0.081 (2)	
H42	0.7195	0.4590	0.2430	0.097*	
C43	0.8081 (6)	0.4928 (3)	0.3516 (5)	0.067 (2)	
C44	0.8058 (6)	0.5070 (3)	0.4330 (5)	0.065 (2)	
H44	0.8662	0.5230	0.4683	0.078*	
C45	0.9072 (7)	0.5032 (4)	0.3212 (7)	0.078 (2)	
C46	0.6529 (6)	1.0083 (3)	0.7105 (5)	0.066 (2)	
C47	0.7441 (6)	0.9768 (3)	0.6899 (4)	0.0484 (16)	
C48	0.7501 (6)	0.9177 (3)	0.6919 (4)	0.0528 (17)	
H48	0.6959	0.8967	0.7069	0.063*	
C49	0.8341 (6)	0.8891 (3)	0.6723 (4)	0.058 (2)	
C50	0.9124 (6)	0.9191 (3)	0.6491 (4)	0.063 (2)	
H50	0.9692	0.8996	0.6357	0.075*	
C51	0.9097 (7)	0.9778 (3)	0.6448 (5)	0.073 (2)	
H51	0.9634	0.9981	0.6281	0.088*	
C52	0.8253 (6)	1.0064 (3)	0.6661 (4)	0.067 (2)	
H52	0.8233	1.0462	0.6642	0.080*	
C53	0.8379 (8)	0.8241 (3)	0.6773 (5)	0.065 (2)	
O1	0.5169 (4)	0.6717 (2)	0.4188 (3)	0.0736 (15)	
O2	0.7106 (4)	0.6477 (2)	0.3893 (3)	0.0721 (15)	
O3	0.9132 (6)	0.6043 (3)	0.9519 (4)	0.114 (2)	
O4	1.1044 (6)	0.6339 (3)	0.9226 (4)	0.117 (2)	
O6	0.9880 (5)	0.5205 (3)	0.3692 (5)	0.108 (2)	
O7	0.6451 (5)	0.4982 (3)	0.5886 (4)	0.105 (2)	
O9	0.6665 (4)	1.0564 (2)	0.7453 (3)	0.0647 (13)	
O10	0.5626 (5)	0.9828 (3)	0.6893 (4)	0.100 (2)	
O11	0.7628 (5)	0.7989 (2)	0.6994 (4)	0.0899 (19)	
O12	0.9136 (5)	0.7986 (2)	0.6586 (4)	0.0844 (17)	
N1	0.6446 (5)	0.6817 (3)	0.6058 (4)	0.0567 (16)	
H1N	0.654 (6)	0.660 (3)	0.566 (3)	0.068*	
N2	0.7561 (4)	0.6687 (2)	0.7885 (3)	0.0499 (14)	
H2N	0.776 (5)	0.6348 (12)	0.789 (4)	0.060*	
N3	0.8784 (5)	0.6731 (3)	0.5614 (4)	0.0558 (15)	
H3N	0.843 (5)	0.6439 (19)	0.547 (4)	0.067*	
N4	0.9934 (5)	0.6679 (3)	0.7413 (4)	0.0573 (16)	
H4N	0.981 (6)	0.649 (3)	0.783 (3)	0.069*	
O5	0.8952 (6)	0.4924 (3)	0.2400 (5)	0.108 (2)	
H5O	0.953 (5)	0.503 (5)	0.228 (7)	0.162*	
O8	0.7893 (5)	0.5506 (3)	0.5885 (3)	0.0796 (16)	
H8O	0.795 (7)	0.549 (4)	0.6431 (17)	0.119*	

### Atomic displacement parameters (Å^2^)

**Table:**

	*U*^11^	*U*^22^	*U*^33^	*U*^12^	*U*^13^	*U*^23^
Cd1	0.0465 (3)	0.0453 (3)	0.0494 (3)	0.0013 (3)	0.0079 (2)	0.0025 (3)
C1	0.066 (5)	0.064 (5)	0.046 (4)	−0.005 (4)	0.005 (4)	−0.009 (4)
C2	0.081 (7)	0.116 (8)	0.074 (6)	0.007 (6)	0.017 (5)	0.011 (6)
C3	0.091 (8)	0.215 (15)	0.070 (7)	−0.046 (9)	0.015 (6)	0.017 (8)
C4	0.150 (12)	0.157 (13)	0.057 (6)	−0.067 (10)	0.017 (8)	0.009 (7)
C5	0.169 (12)	0.087 (8)	0.061 (6)	0.002 (8)	0.002 (7)	0.015 (5)
C6	0.103 (7)	0.067 (6)	0.045 (4)	0.013 (5)	0.004 (5)	0.004 (4)
C7	0.119 (9)	0.145 (10)	0.079 (6)	0.001 (8)	0.009 (7)	0.043 (7)
C8	0.128 (10)	0.103 (9)	0.108 (8)	0.035 (7)	0.008 (7)	0.030 (7)
C9	0.063 (6)	0.114 (8)	0.075 (6)	0.021 (5)	−0.007 (5)	−0.012 (6)
C10	0.087 (7)	0.150 (10)	0.082 (6)	0.051 (7)	−0.033 (6)	−0.036 (6)
C11	0.057 (6)	0.150 (12)	0.145 (11)	0.031 (8)	−0.020 (7)	−0.052 (9)
C12	0.060 (7)	0.126 (10)	0.164 (12)	−0.006 (7)	0.015 (8)	−0.066 (9)
C13	0.057 (5)	0.084 (7)	0.122 (8)	−0.018 (5)	0.007 (5)	−0.029 (5)
C14	0.050 (4)	0.072 (5)	0.072 (5)	0.000 (4)	−0.006 (4)	−0.017 (5)
C15	0.057 (4)	0.056 (4)	0.065 (4)	0.000 (4)	0.005 (4)	−0.009 (4)
C16	0.082 (7)	0.139 (9)	0.098 (7)	0.050 (6)	−0.015 (6)	−0.042 (7)
C17	0.079 (6)	0.175 (11)	0.076 (6)	0.056 (7)	0.024 (5)	0.019 (6)
C18	0.074 (5)	0.049 (4)	0.059 (4)	−0.010 (4)	0.009 (4)	−0.001 (4)
C19	0.066 (5)	0.062 (5)	0.053 (4)	−0.004 (4)	0.016 (4)	0.007 (3)
C20	0.085 (6)	0.086 (7)	0.065 (5)	−0.010 (5)	0.019 (5)	0.004 (4)
C21	0.090 (7)	0.096 (7)	0.084 (6)	−0.007 (6)	0.032 (6)	0.012 (5)
C22	0.125 (8)	0.094 (7)	0.059 (5)	−0.002 (6)	0.041 (6)	−0.002 (5)
C23	0.103 (7)	0.085 (6)	0.048 (4)	−0.014 (5)	0.015 (5)	−0.013 (4)
C24	0.068 (5)	0.062 (5)	0.054 (4)	−0.001 (4)	0.013 (4)	0.005 (4)
C25	0.070 (5)	0.062 (5)	0.065 (5)	−0.007 (4)	−0.005 (4)	−0.013 (4)
C26	0.072 (5)	0.062 (5)	0.078 (5)	−0.006 (4)	0.015 (5)	−0.009 (4)
C27	0.055 (5)	0.064 (5)	0.063 (5)	0.001 (4)	0.007 (4)	0.013 (4)
C28	0.064 (5)	0.067 (6)	0.076 (5)	−0.002 (4)	0.001 (5)	0.007 (4)
C29	0.044 (5)	0.091 (7)	0.119 (8)	0.011 (5)	0.000 (5)	0.023 (6)
C30	0.060 (6)	0.117 (9)	0.111 (8)	0.011 (5)	0.022 (6)	−0.001 (6)
C31	0.066 (5)	0.105 (7)	0.067 (5)	0.024 (5)	0.010 (4)	0.004 (5)
C32	0.062 (5)	0.066 (5)	0.051 (4)	0.013 (4)	0.003 (4)	0.009 (4)
C33	0.059 (4)	0.057 (5)	0.060 (4)	0.004 (4)	0.004 (4)	0.001 (3)
C34	0.059 (5)	0.051 (5)	0.074 (5)	−0.009 (4)	0.002 (4)	0.008 (4)
C35	0.055 (4)	0.061 (5)	0.064 (5)	0.007 (4)	0.017 (4)	0.008 (4)
C36	0.063 (5)	0.058 (5)	0.057 (4)	−0.003 (4)	0.006 (4)	−0.005 (3)
C38	0.077 (6)	0.071 (6)	0.070 (5)	−0.010 (5)	0.023 (5)	0.003 (4)
C39	0.060 (5)	0.053 (5)	0.071 (5)	−0.001 (4)	0.026 (4)	0.010 (4)
C40	0.068 (5)	0.070 (6)	0.085 (6)	−0.017 (4)	0.031 (5)	−0.008 (4)
C41	0.093 (7)	0.087 (7)	0.076 (6)	−0.025 (5)	0.015 (5)	−0.015 (5)
C42	0.102 (7)	0.065 (6)	0.082 (6)	−0.009 (5)	0.039 (6)	−0.007 (4)
C43	0.078 (6)	0.063 (5)	0.069 (5)	−0.001 (4)	0.032 (5)	−0.002 (4)
C44	0.063 (5)	0.052 (5)	0.087 (6)	0.003 (4)	0.032 (4)	0.006 (4)
C45	0.085 (7)	0.071 (6)	0.094 (7)	0.010 (5)	0.054 (6)	0.010 (5)
C46	0.068 (5)	0.056 (5)	0.081 (5)	−0.006 (4)	0.032 (4)	0.003 (4)
C47	0.060 (4)	0.046 (4)	0.044 (4)	0.000 (3)	0.021 (3)	−0.008 (3)
C48	0.063 (5)	0.050 (5)	0.049 (4)	−0.009 (4)	0.020 (4)	−0.003 (3)
C49	0.073 (5)	0.045 (4)	0.047 (4)	−0.002 (4)	−0.005 (4)	−0.002 (3)
C50	0.063 (5)	0.061 (5)	0.065 (5)	0.013 (4)	0.018 (4)	−0.010 (4)
C51	0.085 (6)	0.057 (6)	0.084 (6)	−0.003 (5)	0.034 (5)	0.008 (4)
C52	0.078 (5)	0.049 (4)	0.076 (5)	0.002 (4)	0.024 (5)	0.005 (4)
C53	0.080 (6)	0.050 (5)	0.055 (4)	0.009 (5)	−0.008 (4)	−0.006 (4)
O1	0.074 (4)	0.073 (4)	0.079 (4)	−0.007 (3)	0.027 (3)	−0.011 (3)
O2	0.066 (3)	0.091 (4)	0.058 (3)	−0.013 (3)	0.011 (3)	−0.011 (3)
O3	0.112 (5)	0.157 (7)	0.067 (4)	0.064 (5)	0.007 (4)	0.019 (4)
O4	0.104 (5)	0.155 (7)	0.093 (5)	0.046 (5)	0.027 (4)	0.050 (5)
O6	0.072 (4)	0.138 (6)	0.123 (5)	−0.005 (4)	0.038 (4)	0.008 (5)
O7	0.107 (5)	0.130 (6)	0.093 (4)	−0.040 (4)	0.054 (4)	−0.021 (4)
O9	0.084 (4)	0.043 (3)	0.074 (3)	−0.004 (3)	0.033 (3)	−0.012 (2)
O10	0.083 (4)	0.093 (5)	0.138 (5)	−0.019 (4)	0.052 (4)	−0.039 (4)
O11	0.110 (5)	0.050 (4)	0.104 (4)	0.001 (3)	0.013 (4)	−0.003 (3)
O12	0.090 (4)	0.054 (4)	0.104 (4)	0.018 (3)	0.013 (3)	−0.008 (3)
N1	0.050 (3)	0.063 (4)	0.051 (4)	0.005 (3)	−0.002 (3)	−0.010 (3)
N2	0.047 (3)	0.047 (3)	0.053 (3)	−0.001 (3)	0.006 (3)	−0.004 (3)
N3	0.049 (4)	0.068 (4)	0.050 (3)	0.006 (3)	0.010 (3)	0.008 (3)
N4	0.050 (3)	0.057 (4)	0.058 (4)	0.006 (3)	−0.001 (3)	0.000 (3)
O5	0.130 (6)	0.102 (5)	0.116 (5)	−0.012 (5)	0.075 (5)	−0.006 (4)
O8	0.090 (4)	0.077 (4)	0.068 (3)	−0.022 (3)	0.012 (3)	0.003 (3)

### Geometric parameters (Å, °)

**Table:**

Cd1—N1	2.433 (6)	C26—O1	1.392 (8)
Cd1—N2	2.321 (6)	C26—H26A	0.9700
Cd1—N3	2.329 (6)	C26—H26B	0.9700
Cd1—N4	2.374 (6)	C27—O1	1.370 (9)
Cd1—O11	2.302 (6)	C27—C32	1.387 (10)
Cd1—O12	2.462 (6)	C27—C28	1.401 (10)
Cd1—C53	2.706 (8)	C28—C29	1.369 (11)
C1—C6	1.344 (10)	C28—H28	0.9300
C1—C2	1.388 (11)	C29—C30	1.341 (12)
C1—C36	1.526 (10)	C29—H29	0.9300
C2—C3	1.373 (13)	C30—C31	1.384 (12)
C2—H2	0.9300	C30—H30	0.9300
C3—C4	1.346 (16)	C31—C32	1.376 (10)
C3—H3	0.9300	C31—H31	0.9300
C4—C5	1.360 (15)	C32—C33	1.510 (9)
C4—H4	0.9300	C33—N1	1.487 (8)
C5—C6	1.383 (13)	C33—H33A	0.9700
C5—H5	0.9300	C33—H33B	0.9700
C6—O3	1.375 (10)	C34—N1	1.471 (9)
C7—O3	1.357 (10)	C34—C35	1.509 (9)
C7—C8	1.507 (8)	C34—H34A	0.9700
C7—H7A	0.9700	C34—H34B	0.9700
C7—H7B	0.9700	C35—N2	1.475 (8)
C8—O4	1.343 (11)	C35—H35A	0.9700
C8—H8A	0.9700	C35—H35B	0.9700
C8—H8B	0.9700	C36—N2	1.471 (8)
C9—C14	1.385 (12)	C36—H36A	0.9700
C9—O4	1.391 (11)	C36—H36B	0.9700
C9—C10	1.392 (12)	C38—O7	1.197 (9)
C10—C11	1.376 (15)	C38—O8	1.325 (9)
C10—H10	0.9300	C38—C39	1.478 (10)
C11—C12	1.361 (16)	C39—C44	1.403 (10)
C11—H11	0.9300	C39—C40	1.404 (10)
C12—C13	1.376 (13)	C40—C41	1.386 (10)
C12—H12	0.9300	C40—H40	0.9300
C13—C14	1.351 (11)	C41—C42	1.394 (11)
C13—H13	0.9300	C41—H41	0.9300
C14—C15	1.507 (10)	C42—C43	1.380 (10)
C15—N4	1.475 (9)	C42—H42	0.9300
C15—H15A	0.9700	C43—C44	1.358 (10)
C15—H15B	0.9700	C43—C45	1.492 (8)
C16—N4	1.394 (10)	C44—H44	0.9300
C16—C17	1.506 (8)	C45—O6	1.214 (10)
C16—H16A	0.9700	C45—O5	1.304 (11)
C16—H16B	0.9700	C46—O9	1.246 (8)
C17—N3	1.452 (10)	C46—O10	1.283 (9)
C17—H17A	0.9700	C46—C47	1.490 (10)
C17—H17B	0.9700	C47—C52	1.381 (9)
C18—N3	1.461 (8)	C47—C48	1.382 (9)
C18—C19	1.503 (10)	C48—C49	1.370 (10)
C18—H18A	0.9700	C48—H48	0.9300
C18—H18B	0.9700	C49—C50	1.351 (10)
C19—C20	1.376 (10)	C49—C53	1.518 (10)
C19—C24	1.382 (10)	C50—C51	1.372 (10)
C20—C21	1.379 (11)	C50—H50	0.9300
C20—H20	0.9300	C51—C52	1.385 (10)
C21—C22	1.372 (12)	C51—H51	0.9300
C21—H21	0.9300	C52—H52	0.9300
C22—C23	1.402 (12)	C53—O12	1.239 (9)
C22—H22	0.9300	C53—O11	1.253 (10)
C23—C24	1.371 (10)	N1—H1N	0.85 (6)
C23—H23	0.9300	N2—H2N	0.83 (2)
C24—O2	1.380 (9)	N3—H3N	0.82 (5)
C25—O2	1.420 (8)	N4—H4N	0.85 (6)
C25—C26	1.489 (10)	O5—H5O	0.85 (8)
C25—H25A	0.9700	O8—H8O	0.87 (2)
C25—H25B	0.9700		
			
O11—Cd1—N2	93.5 (2)	C29—C28—H28	120.7
O11—Cd1—N3	127.9 (2)	C27—C28—H28	120.7
N2—Cd1—N3	135.4 (2)	C30—C29—C28	122.0 (9)
O11—Cd1—N4	128.5 (2)	C30—C29—H29	119.0
N2—Cd1—N4	90.1 (2)	C28—C29—H29	119.0
N3—Cd1—N4	77.1 (2)	C29—C30—C31	119.4 (9)
O11—Cd1—N1	88.9 (2)	C29—C30—H30	120.3
N2—Cd1—N1	77.4 (2)	C31—C30—H30	120.3
N3—Cd1—N1	86.6 (2)	C32—C31—C30	121.3 (8)
N4—Cd1—N1	141.5 (2)	C32—C31—H31	119.3
O11—Cd1—O12	54.7 (2)	C30—C31—H31	119.3
N2—Cd1—O12	135.87 (19)	C31—C32—C27	118.2 (7)
N3—Cd1—O12	87.1 (2)	C31—C32—C33	121.7 (7)
N4—Cd1—O12	89.0 (2)	C27—C32—C33	120.0 (7)
N1—Cd1—O12	125.1 (2)	N1—C33—C32	112.7 (6)
C6—C1—C2	118.1 (9)	N1—C33—H33A	109.0
C6—C1—C36	121.7 (8)	C32—C33—H33A	109.0
C2—C1—C36	120.1 (7)	N1—C33—H33B	109.0
C3—C2—C1	120.2 (10)	C32—C33—H33B	109.0
C3—C2—H2	119.9	H33A—C33—H33B	107.8
C1—C2—H2	119.9	N1—C34—C35	111.3 (6)
C4—C3—C2	119.6 (12)	N1—C34—H34A	109.4
C4—C3—H3	120.2	C35—C34—H34A	109.4
C2—C3—H3	120.2	N1—C34—H34B	109.4
C3—C4—C5	121.7 (12)	C35—C34—H34B	109.4
C3—C4—H4	119.2	H34A—C34—H34B	108.0
C5—C4—H4	119.2	N2—C35—C34	111.8 (6)
C4—C5—C6	117.8 (11)	N2—C35—H35A	109.3
C4—C5—H5	121.1	C34—C35—H35A	109.3
C6—C5—H5	121.1	N2—C35—H35B	109.3
C1—C6—O3	115.9 (8)	C34—C35—H35B	109.3
C1—C6—C5	122.4 (10)	H35A—C35—H35B	107.9
O3—C6—C5	121.7 (9)	N2—C36—C1	113.7 (6)
O3—C7—C8	113.3 (8)	N2—C36—H36A	108.8
O3—C7—H7A	108.9	C1—C36—H36A	108.8
C8—C7—H7A	108.9	N2—C36—H36B	108.8
O3—C7—H7B	108.9	C1—C36—H36B	108.8
C8—C7—H7B	108.9	H36A—C36—H36B	107.7
H7A—C7—H7B	107.7	O7—C38—O8	124.1 (8)
O4—C8—C7	109.5 (8)	O7—C38—C39	123.4 (8)
O4—C8—H8A	109.8	O8—C38—C39	112.6 (8)
C7—C8—H8A	109.8	C44—C39—C40	119.0 (7)
O4—C8—H8B	109.8	C44—C39—C38	122.2 (7)
C7—C8—H8B	109.8	C40—C39—C38	118.8 (7)
H8A—C8—H8B	108.2	C41—C40—C39	118.4 (8)
C14—C9—O4	115.9 (8)	C41—C40—H40	120.8
C14—C9—C10	121.2 (11)	C39—C40—H40	120.8
O4—C9—C10	123.0 (10)	C40—C41—C42	121.3 (8)
C11—C10—C9	118.7 (12)	C40—C41—H41	119.3
C11—C10—H10	120.7	C42—C41—H41	119.3
C9—C10—H10	120.7	C43—C42—C41	120.0 (8)
C12—C11—C10	120.5 (11)	C43—C42—H42	120.0
C12—C11—H11	119.7	C41—C42—H42	120.0
C10—C11—H11	119.7	C44—C43—C42	119.4 (8)
C11—C12—C13	119.4 (12)	C44—C43—C45	119.2 (8)
C11—C12—H12	120.3	C42—C43—C45	121.4 (8)
C13—C12—H12	120.3	C43—C44—C39	122.0 (7)
C14—C13—C12	122.4 (11)	C43—C44—H44	119.0
C14—C13—H13	118.8	C39—C44—H44	119.0
C12—C13—H13	118.8	O6—C45—O5	125.9 (8)
C13—C14—C9	117.8 (8)	O6—C45—C43	121.5 (9)
C13—C14—C15	123.1 (8)	O5—C45—C43	112.7 (9)
C9—C14—C15	119.1 (8)	O9—C46—O10	124.3 (8)
N4—C15—C14	113.5 (6)	O9—C46—C47	120.4 (7)
N4—C15—H15A	108.9	O10—C46—C47	115.3 (7)
C14—C15—H15A	108.9	C52—C47—C48	117.6 (7)
N4—C15—H15B	108.9	C52—C47—C46	120.3 (7)
C14—C15—H15B	108.9	C48—C47—C46	122.1 (7)
H15A—C15—H15B	107.7	C49—C48—C47	121.5 (7)
N4—C16—C17	117.0 (7)	C49—C48—H48	119.2
N4—C16—H16A	108.1	C47—C48—H48	119.2
C17—C16—H16A	108.1	C50—C49—C48	119.6 (7)
N4—C16—H16B	108.1	C50—C49—C53	121.0 (8)
C17—C16—H16B	108.1	C48—C49—C53	119.3 (8)
H16A—C16—H16B	107.3	C49—C50—C51	121.2 (7)
N3—C17—C16	112.8 (7)	C49—C50—H50	119.4
N3—C17—H17A	109.0	C51—C50—H50	119.4
C16—C17—H17A	109.0	C50—C51—C52	118.7 (8)
N3—C17—H17B	109.0	C50—C51—H51	120.6
C16—C17—H17B	109.0	C52—C51—H51	120.6
H17A—C17—H17B	107.8	C47—C52—C51	121.3 (7)
N3—C18—C19	113.5 (6)	C47—C52—H52	119.4
N3—C18—H18A	108.9	C51—C52—H52	119.4
C19—C18—H18A	108.9	O12—C53—O11	123.3 (8)
N3—C18—H18B	108.9	O12—C53—C49	118.9 (9)
C19—C18—H18B	108.9	O11—C53—C49	117.8 (8)
H18A—C18—H18B	107.7	O12—C53—Cd1	65.3 (4)
C20—C19—C24	116.8 (7)	O11—C53—Cd1	58.0 (4)
C20—C19—C18	122.7 (7)	C49—C53—Cd1	174.5 (6)
C24—C19—C18	120.5 (7)	C27—O1—C26	117.8 (6)
C19—C20—C21	124.3 (8)	C24—O2—C25	118.1 (6)
C19—C20—H20	117.8	C7—O3—C6	118.3 (7)
C21—C20—H20	117.8	C8—O4—C9	120.1 (8)
C22—C21—C20	117.1 (9)	C53—O11—Cd1	94.5 (5)
C22—C21—H21	121.4	C53—O12—Cd1	87.4 (5)
C20—C21—H21	121.4	C34—N1—C33	113.8 (6)
C21—C22—C23	120.9 (8)	C34—N1—Cd1	104.0 (4)
C21—C22—H22	119.6	C33—N1—Cd1	117.3 (4)
C23—C22—H22	119.6	C34—N1—H1N	105 (5)
C24—C23—C22	119.3 (8)	C33—N1—H1N	112 (5)
C24—C23—H23	120.3	Cd1—N1—H1N	104 (5)
C22—C23—H23	120.3	C36—N2—C35	113.3 (6)
C23—C24—O2	124.0 (7)	C36—N2—Cd1	118.0 (4)
C23—C24—C19	121.6 (8)	C35—N2—Cd1	106.4 (4)
O2—C24—C19	114.4 (7)	C36—N2—H2N	110 (5)
O2—C25—C26	108.2 (6)	C35—N2—H2N	105 (5)
O2—C25—H25A	110.1	Cd1—N2—H2N	103 (5)
C26—C25—H25A	110.1	C17—N3—C18	108.4 (6)
O2—C25—H25B	110.1	C17—N3—Cd1	107.8 (5)
C26—C25—H25B	110.1	C18—N3—Cd1	112.8 (4)
H25A—C25—H25B	108.4	C17—N3—H3N	112 (5)
O1—C26—C25	109.5 (7)	C18—N3—H3N	110 (5)
O1—C26—H26A	109.8	Cd1—N3—H3N	106 (5)
C25—C26—H26A	109.8	C16—N4—C15	116.7 (7)
O1—C26—H26B	109.8	C16—N4—Cd1	106.7 (4)
C25—C26—H26B	109.8	C15—N4—Cd1	114.6 (4)
H26A—C26—H26B	108.2	C16—N4—H4N	116 (5)
O1—C27—C32	116.3 (7)	C15—N4—H4N	99 (5)
O1—C27—C28	123.2 (7)	Cd1—N4—H4N	102 (5)
C32—C27—C28	120.4 (8)	C45—O5—H5O	105 (8)
C29—C28—C27	118.5 (8)	C38—O8—H8O	107 (7)
			
C6—C1—C2—C3	−0.4 (13)	N1—Cd1—C53—O12	−134.0 (4)
C36—C1—C2—C3	177.5 (8)	N2—Cd1—C53—O11	−35.7 (5)
C1—C2—C3—C4	−1.8 (16)	N3—Cd1—C53—O11	140.9 (5)
C2—C3—C4—C5	4.2 (18)	N4—Cd1—C53—O11	−136.5 (5)
C3—C4—C5—C6	−4.2 (17)	N1—Cd1—C53—O11	48.8 (5)
C2—C1—C6—O3	178.8 (7)	O12—Cd1—C53—O11	−177.2 (8)
C36—C1—C6—O3	0.9 (11)	C32—C27—O1—C26	140.3 (7)
C2—C1—C6—C5	0.3 (12)	C28—C27—O1—C26	−42.5 (10)
C36—C1—C6—C5	−177.6 (7)	C25—C26—O1—C27	175.0 (6)
C4—C5—C6—C1	1.9 (14)	C23—C24—O2—C25	−12.8 (11)
C4—C5—C6—O3	−176.5 (9)	C19—C24—O2—C25	168.9 (6)
O3—C7—C8—O4	49.9 (13)	C26—C25—O2—C24	−179.4 (6)
C14—C9—C10—C11	1.2 (15)	C8—C7—O3—C6	−175.5 (9)
O4—C9—C10—C11	−179.2 (9)	C1—C6—O3—C7	137.9 (9)
C9—C10—C11—C12	0.4 (17)	C5—C6—O3—C7	−43.6 (13)
C10—C11—C12—C13	−1.1 (18)	C7—C8—O4—C9	171.2 (8)
C11—C12—C13—C14	0.1 (15)	C14—C9—O4—C8	163.9 (9)
C12—C13—C14—C9	1.5 (13)	C10—C9—O4—C8	−15.7 (14)
C12—C13—C14—C15	−176.5 (8)	O12—C53—O11—Cd1	−3.1 (8)
O4—C9—C14—C13	178.3 (7)	C49—C53—O11—Cd1	176.0 (5)
C10—C9—C14—C13	−2.2 (13)	N2—Cd1—O11—C53	148.4 (5)
O4—C9—C14—C15	−3.6 (11)	N3—Cd1—O11—C53	−49.4 (6)
C10—C9—C14—C15	175.9 (8)	N4—Cd1—O11—C53	55.5 (6)
C13—C14—C15—N4	108.1 (9)	N1—Cd1—O11—C53	−134.2 (5)
C9—C14—C15—N4	−69.9 (9)	O12—Cd1—O11—C53	1.6 (4)
N4—C16—C17—N3	−52.6 (13)	O11—C53—O12—Cd1	2.9 (8)
N3—C18—C19—C20	−101.1 (8)	C49—C53—O12—Cd1	−176.2 (6)
N3—C18—C19—C24	81.9 (9)	O11—Cd1—O12—C53	−1.6 (4)
C24—C19—C20—C21	0.5 (12)	N2—Cd1—O12—C53	−53.2 (5)
C18—C19—C20—C21	−176.6 (8)	N3—Cd1—O12—C53	140.5 (5)
C19—C20—C21—C22	0.9 (13)	N4—Cd1—O12—C53	−142.4 (5)
C20—C21—C22—C23	−0.9 (14)	N1—Cd1—O12—C53	56.7 (5)
C21—C22—C23—C24	−0.5 (13)	C35—C34—N1—C33	−85.7 (7)
C22—C23—C24—O2	−176.2 (7)	C35—C34—N1—Cd1	43.0 (6)
C22—C23—C24—C19	2.1 (12)	C32—C33—N1—C34	−53.0 (8)
C20—C19—C24—C23	−2.0 (11)	C32—C33—N1—Cd1	−174.6 (5)
C18—C19—C24—C23	175.1 (7)	O11—Cd1—N1—C34	−108.0 (5)
C20—C19—C24—O2	176.4 (6)	N2—Cd1—N1—C34	−14.1 (4)
C18—C19—C24—O2	−6.5 (10)	N3—Cd1—N1—C34	124.0 (4)
O2—C25—C26—O1	56.7 (8)	N4—Cd1—N1—C34	59.7 (6)
O1—C27—C28—C29	−176.8 (7)	O12—Cd1—N1—C34	−152.0 (4)
C32—C27—C28—C29	0.3 (11)	C53—Cd1—N1—C34	−128.3 (5)
C27—C28—C29—C30	2.4 (13)	O11—Cd1—N1—C33	18.6 (5)
C28—C29—C30—C31	−2.4 (15)	N2—Cd1—N1—C33	112.5 (5)
C29—C30—C31—C32	−0.3 (14)	N3—Cd1—N1—C33	−109.4 (5)
C30—C31—C32—C27	2.9 (12)	N4—Cd1—N1—C33	−173.7 (4)
C30—C31—C32—C33	−172.6 (8)	O12—Cd1—N1—C33	−25.4 (6)
O1—C27—C32—C31	174.4 (7)	C53—Cd1—N1—C33	−1.7 (6)
C28—C27—C32—C31	−2.9 (11)	C1—C36—N2—C35	67.6 (8)
O1—C27—C32—C33	−10.0 (10)	C1—C36—N2—Cd1	−167.2 (5)
C28—C27—C32—C33	172.7 (6)	C34—C35—N2—C36	177.0 (6)
C31—C32—C33—N1	108.6 (8)	C34—C35—N2—Cd1	45.8 (6)
C27—C32—C33—N1	−66.9 (9)	O11—Cd1—N2—C36	−56.5 (5)
N1—C34—C35—N2	−64.3 (8)	N3—Cd1—N2—C36	143.7 (4)
C6—C1—C36—N2	83.3 (8)	N4—Cd1—N2—C36	72.0 (5)
C2—C1—C36—N2	−94.5 (8)	N1—Cd1—N2—C36	−144.6 (5)
O7—C38—C39—C44	−164.4 (8)	O12—Cd1—N2—C36	−16.7 (6)
O8—C38—C39—C44	15.6 (11)	C53—Cd1—N2—C36	−40.9 (5)
O7—C38—C39—C40	16.9 (13)	O11—Cd1—N2—C35	72.1 (4)
O8—C38—C39—C40	−163.2 (7)	N3—Cd1—N2—C35	−87.7 (5)
C44—C39—C40—C41	0.2 (11)	N4—Cd1—N2—C35	−159.4 (4)
C38—C39—C40—C41	179.1 (8)	N1—Cd1—N2—C35	−16.0 (4)
C39—C40—C41—C42	−0.9 (13)	O12—Cd1—N2—C35	111.9 (4)
C40—C41—C42—C43	1.0 (13)	C53—Cd1—N2—C35	87.7 (4)
C41—C42—C43—C44	−0.4 (12)	C16—C17—N3—C18	159.4 (8)
C41—C42—C43—C45	179.3 (8)	C16—C17—N3—Cd1	37.0 (10)
C42—C43—C44—C39	−0.4 (12)	C19—C18—N3—C17	70.6 (8)
C45—C43—C44—C39	180.0 (7)	C19—C18—N3—Cd1	−170.1 (5)
C40—C39—C44—C43	0.4 (11)	O11—Cd1—N3—C17	114.7 (6)
C38—C39—C44—C43	−178.4 (7)	N2—Cd1—N3—C17	−91.1 (6)
C44—C43—C45—O6	5.1 (13)	N4—Cd1—N3—C17	−14.4 (6)
C42—C43—C45—O6	−174.6 (9)	N1—Cd1—N3—C17	−159.3 (6)
C44—C43—C45—O5	−174.3 (8)	O12—Cd1—N3—C17	75.3 (6)
C42—C43—C45—O5	6.0 (11)	C53—Cd1—N3—C17	93.1 (6)
O9—C46—C47—C52	−33.8 (10)	O11—Cd1—N3—C18	−4.9 (6)
O10—C46—C47—C52	145.5 (7)	N2—Cd1—N3—C18	149.3 (4)
O9—C46—C47—C48	147.6 (7)	N4—Cd1—N3—C18	−134.0 (5)
O10—C46—C47—C48	−33.0 (10)	N1—Cd1—N3—C18	81.1 (5)
C52—C47—C48—C49	1.0 (10)	O12—Cd1—N3—C18	−44.3 (4)
C46—C47—C48—C49	179.6 (6)	C53—Cd1—N3—C18	−26.5 (5)
C47—C48—C49—C50	−1.1 (10)	C17—C16—N4—C15	−94.1 (9)
C47—C48—C49—C53	178.7 (6)	C17—C16—N4—Cd1	35.4 (10)
C48—C49—C50—C51	0.1 (11)	C14—C15—N4—C16	−51.4 (9)
C53—C49—C50—C51	−179.7 (7)	C14—C15—N4—Cd1	−177.1 (5)
C49—C50—C51—C52	0.9 (11)	O11—Cd1—N4—C16	−139.1 (6)
C48—C47—C52—C51	0.0 (10)	N2—Cd1—N4—C16	126.3 (6)
C46—C47—C52—C51	−178.6 (7)	N3—Cd1—N4—C16	−10.5 (6)
C50—C51—C52—C47	−1.0 (11)	N1—Cd1—N4—C16	56.7 (7)
C50—C49—C53—O12	−1.0 (10)	O12—Cd1—N4—C16	−97.8 (6)
C48—C49—C53—O12	179.2 (6)	C53—Cd1—N4—C16	−115.1 (6)
C50—C49—C53—O11	179.9 (7)	O11—Cd1—N4—C15	−8.3 (6)
C48—C49—C53—O11	0.1 (10)	N2—Cd1—N4—C15	−102.9 (5)
O11—Cd1—C53—O12	177.2 (8)	N3—Cd1—N4—C15	120.2 (5)
N2—Cd1—C53—O12	141.4 (4)	N1—Cd1—N4—C15	−172.5 (4)
N3—Cd1—C53—O12	−41.9 (5)	O12—Cd1—N4—C15	33.0 (5)
N4—Cd1—C53—O12	40.6 (5)	C53—Cd1—N4—C15	15.7 (6)

### Hydrogen-bond geometry (Å, °)

**Table:**

*D*—H···*A*	*D*—H	H···*A*	*D*···*A*	*D*—H···*A*
N3—H3N···O8	0.82 (5)	2.42 (4)	3.148 (8)	147 (6)
N4—H4N···O4	0.85 (6)	2.46 (6)	3.043 (8)	127 (6)
N2—H2N···O9^i^	0.83 (2)	2.10 (3)	2.901 (7)	163 (6)
O8—H8O···O9^i^	0.87 (2)	1.76 (3)	2.605 (7)	166 (9)
O5—H5O···O10^ii^	0.85 (8)	1.71 (8)	2.548 (9)	170 (12)

Symmetry codes: (i) −*x*+3/2, *y*−1/2, −*z*+3/2; (ii) *x*+1/2, −*y*+3/2, *z*−1/2.
